# Evaluating the Positive Functioning at Work (PF-W) Questionnaire: Insights into Predictive Factors of Well-Being Among Spanish Workers

**DOI:** 10.3390/bs15040455

**Published:** 2025-04-02

**Authors:** Adrián García-Selva, Marie-Carmen Neipp, Beatriz Martín-del-Río

**Affiliations:** 1 Department of Behavioral and Health Sciences, Miguel Hernández University of Elche, 03202 Elche, Alicante, Spain; adrian.garcias@umh.es; 2 Department of Health Psychology, Miguel Hernández University of Elche, 03202 Elche, Alicante, Spain; neipp@umh.es

**Keywords:** workplace well-being, PERMA+4 model, self-efficacy, perceived organizational support, positive emotions, job satisfaction, organizational commitment, structural equation modeling

## Abstract

This study examines the interplay between individual predictors (self-efficacy) and organizational factors (perceived organizational support) within the framework of the PERMA+4 model to promote workplace well-being. Data were collected from 545 employees (57.8% women) using self-reported questionnaires and analyzed through structural equation modeling. The results indicate that self-efficacy positively influences seven dimensions of the PERMA+4 model, while perceived organizational support significantly affects five dimensions. Positive emotions are identified as a mediator, amplifying the impact of PERMA+4 dimensions on job satisfaction and organizational commitment. Specifically, positive emotions have a positive effect on job satisfaction, underscoring their pivotal role in the workplace well-being. These findings validate the PERMA+4 model as a comprehensive framework for understanding workplace well-being, emphasizing the dynamic interaction between individual and organizational factors. Moreover, they provide actionable insights for interventions aimed at enhancing employee satisfaction and long-term commitment by fostering self-efficacy, organizational support, and positive emotions.

## 1. Introduction

The mainstream issues in work and organizational psychology have become work well-being and positive functioning. They represent the interaction of individual, social, and organizational factors related to the satisfaction and performance of employees. Besides the effect on productivity, employee well-being is associated with organizational benefits such as a healthy organizational climate, stress reduction, commitment by employees, and performance in the organization, among many others (Donaldson & Ko, 2010; Rothmann, 2013). Some of the well-being models at work that now allow for a fuller understanding are the PERMA+4 model. Donaldson and colleagues (2020) introduce the PERMA+4 framework to evaluate well-being in the workplace. This model includes nine key components of well-being, five of which are based on Seligman’s (2011) original framework, as previously mentioned, with four additional elements designed to enhance the PERMA model within a work setting. These four new elements were developed following extensive research (Donaldson & Ko, 2010; Donaldson et al., 2019), which analyzed empirical studies using the PERMA-profiler in the workplace, along with a comprehensive review and meta-analysis of positive psychology interventions at work. Their findings suggested that, alongside Seligman’s five components, these four additional factors could significantly contribute to better well-being and job performance. The four new elements are: Physical Health, Mindset (fostering a future-oriented and growth mindset), Environment (the quality of the physical workspace, including access to natural light and nature), and Economic Security (the sense of financial stability). Cabrera and Donaldson (2024) found strong evidence that the key elements and overall structure of PERMA and PERMA+4 help predict well-being and positive work performance, making them promising for future use and interventions.

From this point of view, both individual capacities and organization dynamics contribute to better work ability, that is, enhancing productivity, supporting physical and mental well-being, and facilitating effective adaptation to workplace demands over time.

In that line, the PERMA+4 dimensions cannot only represent the main factors of well-being at work, but also play a mediational role in variables such as self-efficacy and POS, and outcomes of well-being at work, such as job satisfaction and organizational commitment. In this regard, job satisfaction, that is, a positive evaluation of work and its conditions (Furnham et al., 2009), may follow from the very fact of the interaction of the PERMA+4 dimensions. Likewise, organizational commitment can be assumed to be an emotional and psychological identification of an employee with his or her organization (Luthans, 2002), which could be influenced by job satisfaction, POS, and a growth mindset, ensuring a sense of belonging and loyalty toward the organization.

The aim of this study is to examine how various predictive factors, such as self-efficacy and perceived organizational support, interact with the nine dimensions of the PERMA+4 model and, through these interactions, influence key outcomes like job satisfaction and organizational commitment. While previous research has explored the individual impact of these variables, there is still limited empirical evidence on how they jointly contribute to well-being in organizational contexts. By addressing this gap, the present study seeks not only to enhance our understanding of the mechanisms that sustain well-being at work, but also to provide an empirical foundation for designing effective, evidence-based interventions aimed at promoting employee flourishing in the workplace.

### 1.1. Literature Review

#### 1.1.1. Self-Efficacy

Among the individual capacities, self-efficacy (Bandura, 1997) is defined as a person’s belief in his own capacities to perform in a particular situation or to solve specific problems at his workplace. This attitude is very relevant for the improvement of well-being. It is a key psychological resource for strengthening the dimensions of the PERMA+4 model and fostering a work environment where individuals can reach their full potential (Cabrera, 2024; Donaldson & Donaldson, 2021b). This concept is a core component of Psychological Capital (PsyCap), which encompasses psychological resources such as resilience, hope, and optimism, all of which are critical for workplace adaptation and performance (Luthans et al., 2007). Individuals with high levels of PsyCap tend to develop a Positive Mindset that enables them to overcome adversity and seize opportunities for personal and professional growth, thereby enhancing their work engagement, interpersonal relationships, and sense of accomplishment (Gao et al., 2023; Martin & Donaldson, 2024; Vogelgesang et al., 2014).

Recent research has demonstrated that interventions designed to enhance PsyCap, including self-efficacy, are effective and sustainable across various cultures and organizational contexts. These interventions not only promote personal well-being, but also strengthen organizational cohesion and performance (Lupsa et al., 2019; Salanova & Ortega-Maldonado, 2019).

The PERMA+4 model provides a structured framework for understanding workplace well-being, integrating both individual and organizational factors. The interaction between PsyCap and the pillars of the model reinforces aspects such as adaptability and work meaning, promoting positive and productive work environments (Cabrera & Donaldson, 2024; Da et al., 2020; Weiss et al., 2024). This impact extends beyond the individual, benefiting the collective through the creation of a supportive and resilient environment.

Thus, the following is proposed:

**Hypothesis 1:**  *Self-efficacy will have positive effects on the PERMA+4 dimensions.*

#### 1.1.2. Organizational Support

Perceived Organizational Support (POS) is defined as the degree to which workers feel that the organization cares for them and respects them as people (Eisenberger et al., 1990). Organizational support is a fundamental driver of workplace well-being. When employees feel that their contributions are valued and their well-being matters, their work experience (well-being, motivation, performance) transforms (Cabrera, 2024; Cabrera & Donaldson, 2024; Mihalache & Mihalache, 2022; Weiss et al., 2024). Leadership that fosters trust, mentorship, and professional development not only enhances individual well-being, but also strengthens key dimensions of the PERMA+4 model, such as personal accomplishment, positive emotions, and meaningful relationships (Donaldson et al., 2024a).

Beyond individual benefits, perceived organizational support also fosters collaboration and commitment within teams, contributing to a more resilient and productive work environment (Donaldson et al., 2023). Empirical research highlights that workplaces characterized by emotional support and positive interpersonal interactions enable employees to cultivate meaningful relationships and experience positive emotions, both of which are integral components of the PERMA+4 framework (Donaldson et al., 2024a; Mihalache & Mihalache, 2022).

Thus, the following is proposed:

**Hypothesis 2:**  *Organizational support will have positive effects on the PERMA+4 dimensions.*

Additionally, organizational support is not only a facilitator of individual well-being, but also a key strategy for improving job satisfaction and, ultimately, organizational success. Employees who perceive strong organizational support—particularly when their efforts are recognized, and their professional growth is encouraged—develop a deeper emotional connection to their work (Donaldson et al., 2024a). This sense of connection enhances feelings of accomplishment and contributes to a greater sense of stability and purpose in the workplace (Mihalache & Mihalache, 2022). Research has shown that organizational practices emphasizing trust, mentorship, and employee well-being significantly contribute to job satisfaction. Employees in supportive environments tend to display higher levels of motivation, engagement, and resilience in response to workplace challenges (Donaldson et al., 2024a; Rasool et al., 2021).

Thus, the following is proposed:

**Hypothesis 3:**  *Organizational support will have positive effects on Job Satisfaction.*

Organizational commitment extends beyond employee retention; it reflects the depth of an individual’s emotional attachment and identification with their organization. Employees who perceive high levels of organizational support tend to develop stronger loyalty and alignment with their company’s mission and values (Cabrera, 2024; Donaldson et al., 2024a; Hngoi et al., 2024). A workplace culture that prioritizes employee well-being not only strengthens interpersonal relationships, but also fosters a climate of trust, which is essential for cultivating long-term commitment (Donaldson et al., 2024a; Zagenczyk et al., 2020).

Thus, the following is proposed:

**Hypothesis 4:**  *Organizational support will have positive effects on Organizational Commitment.*

#### 1.1.3. Workplace Well-Being

Workplace well-being is deeply influenced by the interaction of multiple organizational and personal factors. Positive emotions not only reflect high-quality work experiences, but also naturally emerge when employees experience job satisfaction, accomplishment, and organizational support (Wall et al., 2021).

Among the key drivers of positive emotions at work are high-quality workplace relationships, which provide a strong sense of support and belonging (Donaldson et al., 2024a; Dutton, 2003). Furthermore, employees who find meaning in their work and achieve personal or professional goals experience increased satisfaction and motivation, reinforcing their self-esteem (Krauss & Orth, 2022). Likewise, economic security and a stable work environment reduce stress and enhance emotional well-being (Wall et al., 2021).

Thus, the following is proposed:

**Hypothesis 5:**  *The PERMA+4 dimensions of Engagement, Relationships, Meaning, Accomplishment, Physical Health, Mindset, Environment, and Economic Security will have positive effects on the PERMA+4 dimension of Positive Emotions.*

The PERMA+4 model also provides a comprehensive framework explaining how dimensions such as engagement, relationships, meaning, accomplishment, physical health, mindset, environment, and economic security positively impact job satisfaction and organizational commitment. Research has shown that job satisfaction is closely linked to the elements of the model. Engagement, for instance, creates states of flow that enhance satisfaction (Weintraub et al., 2021). Similarly, positive workplace relationships, characterized by support and trust, strengthen employees’ sense of belonging and significantly improve their perception of job satisfaction (Bulińska-Stangrecka & Bagieńska, 2021). Another key factor is the perceived meaning of work, which enhances their emotional well-being and reinforces job satisfaction (Donaldson et al., 2024a). Additionally, economic security and a favorable work environment, which foster positive emotions and reduce stress, contribute to satisfaction by providing stability and a setting conducive to personal and professional growth (Donaldson et al., 2020).

On the other hand, organizational commitment is also strengthened by various dimensions of PERMA+4. Workplace relationships based on support and collaboration reinforce employees’ sense of community and commitment to organizational values (Donaldson et al., 2024a; Fredrickson, 2001). Furthermore, achieving meaningful work goals generates a sense of pride and purpose, which strengthens employees’ emotional connection to the organization and promotes long-term commitment (Donaldson & Villalobos, 2024). Additionally, the dimensions of physical health and mindset create more resilient and optimistic employees, that are prepared to align with organizational objectives (Donaldson et al., 2024a).

Recent studies have shown that the dimensions of PERMA+4 do not operate in isolation, but interact to amplify both job satisfaction and organizational commitment. Donaldson et al. (2023) suggest that these dimensions work synergistically to create an environment where employees thrive, contributing to organizational success. Furthermore, positive emotions, which arise as a result of these dimensions, mediate the relationship between PERMA+4 and organizational outcomes, strengthening the connection between employees and the organization (Donaldson et al., 2020).

Thus, the following is proposed:

**Hypothesis 6:**  *The PERMA+4 dimensions of Engagement, Relationships, Meaning, Accomplishment, Physical Health, Mindset, Environment, and Economic Security will have positive effects on Job Satisfaction and Organizational Commitment.*

Positive emotions not only directly contribute to job satisfaction and organizational commitment, but also act as mediators that amplify overall well-being. According to Fredrickson (2001), these emotions expand employees’ cognitive and behavioral resources, helping them navigate workplace challenges more effectively and fostering a sense of emotional and professional fulfillment (Fredrickson, 2001; Garland et al., 2010).

Positive emotions such as joy, hope, and gratitude have been identified as key predictors of job satisfaction. Recent meta-analyses demonstrate that positive affect significantly correlates with job satisfaction, underscoring its central role in workplace well-being (Connolly & Viswesvaran, 2000; Donaldson et al., 2019). Moreover, longitudinal studies have shown that positive emotions explain a significant portion of variability in job satisfaction, even when controlling for other factors (Donaldson & Villalobos, 2024). In healthcare contexts, positive emotions have been identified as one of the greatest contributors to workplace satisfaction (Lanham et al., 2012).

These emotions foster employees’ perceptions of support and satisfaction in the workplace, strengthening emotional connection to the organization (Cabrera, 2024; Kuang et al., 2022; Weiss et al., 2024). Specific components like hope and resilience, which are part of positive emotions, play a key role in building this loyalty (Donaldson et al., 2021). This process not only enhances employees’ capacity to face workplace challenges, but also strengthens their commitment to organizational goals and values (Fredrickson, 2001; Garland et al., 2010).

Thus, the following is proposed:

**Hypothesis 7:**  *Positive Emotions will have positive effects on Job Satisfaction and Organizational Commitment.*

Furthermore, positive emotions are fundamental to organizational well-being, acting not only as an outcome of favorable workplace conditions, but also as a key mediator linking the dimensions of the PERMA+4 model to outcomes such as job satisfaction and organizational commitment.

Positive emotions, such as joy, pride, and gratitude, act as a bridge connecting key elements of PERMA+4 (e.g., meaning, accomplishment, and relationships) to job satisfaction and commitment. Donaldson et al. (2023) found that these emotions mediate the relationship between PERMA+4 dimensions and job satisfaction, explaining a significant proportion of variability in this outcome (Donaldson et al., 2023). Additionally, meaning and accomplishment generate positive emotions that subsequently predict life and work satisfaction (Donaldson & Villalobos, 2024). Fredrickson’s (2001) Broaden-and-Build Theory provides a theoretical framework to understand how positive emotions expand employees’ cognitive and behavioral resources, enabling them to navigate workplace challenges more effectively. This mediating process not only enhances individual well-being, but also strengthens organizational commitment (Fredrickson, 2001).

Longitudinal studies have shown that positive emotions derived from meaning and economic security mediate their relationships with job satisfaction (Donaldson et al., 2020). Research has also identified a bidirectional effect between positive emotions and job satisfaction. Donaldson et al. (2024a) highlight that job satisfaction predicts positive emotions, which in turn reinforce satisfaction, consolidating their role as mediators in workplace well-being. Furthermore, positive workplace relationships generate feelings of support and belonging, fostering positive emotions that strengthen commitment to the organization (Fredrickson, 2001; Donaldson et al., 2024b).

Thus, the following is proposed:

**Hypothesis 8:**  *Positive Emotions mediate the relationship between PERMA+4 dimensions and Job Satisfaction and Organizational Commitment.*

## 2. Materials and Methods

### 2.1. Data Collection and Participants

This study employed a cross-sectional online questionnaire design, with data collected through the Google Form online survey platform. A questionnaire link was generated online, inviting users to complete the survey. Data collection utilized the snowball sampling technique.

In this study participated 545 individuals who were actively employed in the Spanish labor market at the time of responding to the survey, of whom 315 (57.8%) were women and 230 (42.2%) were men. In terms of age, the mean value was 37.07 (*SD* = 13.42), with a minimum of 18 and a maximum of 64. Regarding job level, 451 participants (82.8%) belonged to the basic level, while 67 (12.3%) were middle managers and 27 (5.0%) held managerial positions. Continuing with the distribution by sector, 215 participants (39.4%) belonged to the service sector, 90 (16.5%) were part of the industry sector, 67 (12.3%) belonged to the education sector, 77 (14.1%) worked in commerce, 53 (9.7%) belonged to the health sector, and 43 (7.90%) worked in public administration. Finally, 45 people (8.3%) had basic education, 115 (21.1%) had secondary education, 129 (23.7%) had a vocational training degree, 177 (32.5%) had undergraduate or graduate studies, and 79 (14.5%) had postgraduate studies.

### 2.2. Instruments and Measures

**Figure 1:**
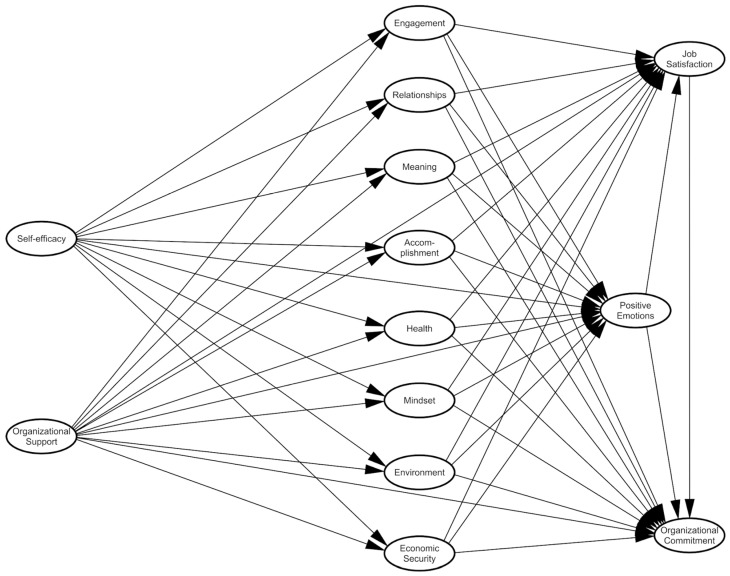
Proposed conceptual model.

According to the conceptualized theoretical framework, there are five main scales in the hypothetical model, as shown in Figure 1. First of all, PERMA+4 was measured through the Positive Functioning at Work Scale developed by Donaldson and Donaldson (2021b) and adapted to the Spanish sample by García-Selva et al. (2024). The scale is composed of 29 items grouped into nine dimensions, with a Likert-type response ranging from 1 (“strongly disagree”) to 7 (“strongly agree”). Following the PERMA framework (Seligman, 2011), Donaldson et al. (2020) adapted the original five factors to the work environment, extending the model with four additional factors (physical health, mindset, environment, and economic security). Thus, the dimensions of this scale are as follows: (1) positive emotions (3 items, e.g., “I feel happy on a typical work day”), (2) engagement (3 items, e.g., “when I am working on something I like, I forget about everything around me”), (3) relationships (4 items, e.g., “I feel valued by my coworkers”), (4) meaning (3 items, e.g., “I understand what makes my work meaningful”), (5) accomplishment (3 items, e.g., “I set goals that help me achieve my professional aspirations”), (6) physical health (4 items, e.g., “in general, I feel physically healthy”), (7) mindset (3 items, e.g., “I believe that my job will allow me to develop in the future”), (8) environment (3 items, e.g., “there is a lot of natural light in my workplace”), and (9) economic security (3 items, e.g., “I feel comfortable with my current income”).

Self-efficacy was assessed with the brief general self-efficacy scale (GSE-3) (Doll et al., 2021), adapting this instrument to the work environment to meet the objectives of the present research. This scale is composed of three items (“I am confident in my own abilities in difficult situations at work”, “I am able to solve most of the problems in my job by myself”, “I can usually solve even difficult and complex tasks in my job well”). The three items have a Likert-type response scale ranging from 1 (“do not agree at all”) to 5 (“strongly agree”).

Perceived organizational support was evaluated using the scale developed by Eisenberger et al. (1990), which consists of four items that measure the degree of support that a worker perceives from his or her direct supervisor (e.g., “my supervisor is concerned about the well-being of his or her workers”, “I feel appreciated by my supervisor”). All items have a Likert-type response with a gradation ranging from 1 (“strongly disagree”) to 5 (“strongly agree”).

Job satisfaction was assessed through the questionnaire developed by the PSYCONES research team (Estreder et al., 2006; Guest et al., 2010; Peiró et al., 2007), adapted from Price’s (1997) instrument, which assesses job satisfaction through four items (e.g., “I enjoy my job”, “most days I am enthusiastic about my job”). The response scale is Likert-type with a range from 1 (“strongly disagree”) to 5 (“strongly agree”).

Finally, organizational commitment was measured using the questionnaire developed by Cook and Wall (1980), composed of five items (e.g., “I feel part of this company”, “even if this organization did not do well, I would be reluctant to change organizations”). The response scale is Likert-type, ranging from 1 (“strongly disagree”) to 5 (“strongly agree”).

### 2.3. Data Analysis

Due to the latent nature of the variables under study, structural equation modeling (SEM) was used to answer the stated hypotheses, due to its ability to simultaneously analyze complex relationships between latent variables, allowing for the assessment of measurement error and construct validity (R. B. Kline, 2023). However, it should be noted that, in research practice in the field of Work and Organizational Psychology, it is common for empirical works to measure multiple constructs with a single method and multiple indicators, thus being within the so-called multitrait-monomethod context (Cheung et al., 2023). Therefore, before testing the stated hypotheses, a confirmatory factor analysis (CFA) was performed to assess the reliability and validity of the measurement model independently of the structural model. Thus, the reliability of the constructs was assessed using the composite reliability (CR) index, whose value is considered appropriate when it is greater than 0.70 (Hair et al., 2019). Convergent validity was assessed through the factor loadings of the items in each factor, and through the average variance extracted (AVE). Regarding factor loadings, the convergent validity of the model is acceptable when all standardized loadings are statistically significant and above 0.50, and ideally above 0.70 (Hair et al., 2019). As for the average variance extracted, this index is considered adequate when it acquires values above 0.50 (Fornell & Larcker, 1981). Regarding discriminant validity, its evaluation was carried out through the comparison of the square root of the average variance extracted with the latent correlations between constructs, through the comparison of the values of the average variance extracted with the values of the maximum shared variance (MSV), and through the heterotrait–monotrait ratio (HTMT2) (Henseler et al., 2015; Roemer et al., 2021). Regarding the latter, HTMT2 construct correlation values should be below 0.90 to provide evidence of discriminant validity (Hair et al., 2019). Regarding the comparison of the square root of the average variance extracted with the correlations between constructs, the former index must be higher than the correlation of the latent constructs to support the existence of discriminant validity (Hair et al., 2019). Finally, regarding the comparison of the average variance values extracted with the maximum shared variance values, again, the mean variance extracted must be greater than the maximum shared variance to support the existence of discriminant validity (Hair et al., 2019).

To evaluate the fit of the models, the guidelines proposed by Brown (2015) were followed and the next indices were used: chi-square ratio over degrees of freedom (χ^2^/*df*), comparative fit index (CFI), Tucker-Lewis index (TLI), root mean square error of approximation (RMSEA), and standardized root mean square residual (SRMR). The χ^2^ ratio over the degrees of freedom should obtain values less than 3 to indicate a good fit. Regarding the CFI and TLI, values above 0.95 are preferable for these indices, while values close to 0.90 are considered acceptable. For its part, the RMSEA value should be less than 0.08 to determine a reasonable fit, and less than 0.05 to indicate an optimal fit. Finally, values below 0.08 for the SRMR indicate a good model fit.

To assess the practical significance (i.e., the effect size of the relationships observed among the latent constructs), the magnitude of the standardized path coefficient (β) was evaluated, using the standards recommended by T. J. Kline (2005): a β value greater than |0.10| indicates a small effect size, a β value greater than |0.30| indicates a medium effect size, and a β value greater than |0.50| indicates a large effect size. In addition, another practical significance measure was used, which corresponds to the amount of explained variance (*R*^2^) on dependent variables accounted for by the hypothesized model. This index was evaluated using the threshold values proposed by Hair et al. (2011): an *R*^2^ > 0.25 indicates a small effect size, an *R*^2^ > 0.50 suggests a medium effect size, and an *R*^2^ ≥ 0.75 is taken as a large effect size.

Lastly, to evaluate the mediation effects (indirect effects), the Monte Carlo method (MacKinnon et al., 2004) was used, with 10,000 bootstrap samples with a 95% confidence interval. Thus, when the confidence interval of the indirect effect does not include zero, the effect is statistically significant.

All analyses were carried out using the packages “lavaan” (Rosseel, 2012) and “semTools” (Jorgensen et al., 2022) in R (version 4.3.0). Maximum likelihood estimation with robust standard errors (MLRs) (Yuan & Bentler, 2000) was used for the CFA and the structural equation model, due to the number of categories used for each item of each variable (Rhemtulla et al., 2012).

## 3. Results

The results of the CFA indicated a very adequate fit between the measurement model and the data (χ^2^/*df* = 2.023; CFI = 0.928; TLI = 0.917; RMSEA = 0.048; SRMR = 0.054). All standardized factor loadings of the model were statistically significant and above 0.50, while the coefficients corresponding to the Cronbach’s alphas of each dimension were above 0.70 (Table 1). Moreover, as can be seen in Table 2, the composite reliability coefficients of each latent factor were above 0.70, and the values of the average variance extracted (AVE) ranged from 0.503 (commitment) to 0.760 (positive emotions). These results guarantee the reliability and convergent validity of the measurement model. As for discriminant validity, the values corresponding to the square root of the average variance extracted were always higher than the correlation indices of the latent variables, and the average variance extracted was always greater than the maximum shared variance (MSV), which supports the existence of discriminant validity between the constructs (Table 2).

On the other hand, Table 3 shows the results of the heterotrait–monotrait ratio (HTMT2). In this case, similar to the previous results and providing support for the presence of discriminant validity, all HTMT2 correlation values were below 0.90 (Hair et al., 2019).

**Figure 2:**
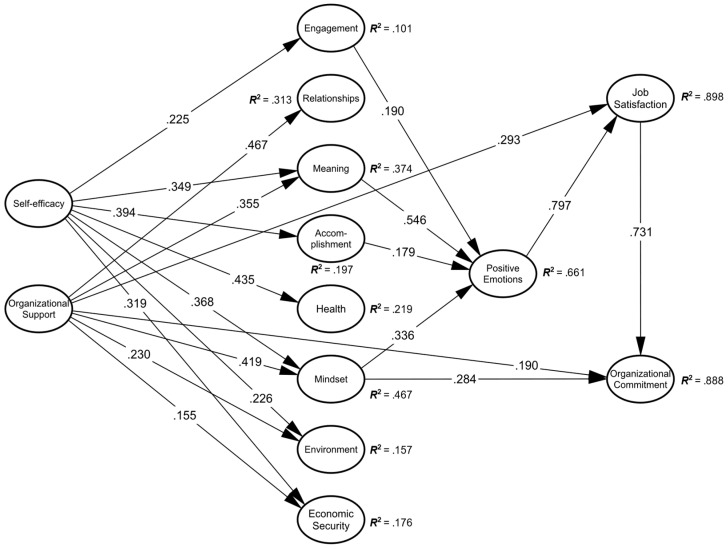
Results of the structural equation model (non-significant regressions between latent variables have been omitted).

Once the reliability and convergent and discriminant validity of the variables analyzed in the model had been verified, the hypotheses were tested using structural equation modeling. In this regard, the results indicate that the structural model fitted the data correctly (χ^2^/*df* = 2.236; CFI = 0.910; TLI = 0.901; RMSEA = 0.053; SRMR = 0.076). The analysis confirmed the existence of positive relationships between self-efficacy and organizational support with the PERMA+4 dimensions (Figure 2). Specifically, self-efficacy positively and significantly influenced engagement (β = 0.225, *p* = 0.022), meaning (β = 0.349, *p* = 0.002), accomplishment (β = 0.394, *p* = 0.001), physical health (β = 0.435, *p* = 0.000), mindset (β = 0.368, *p* = 0.003), environment (β = 0.226, *p* = 0.023), and economic security (β = 0.319, *p* = 0.000). For its part, organizational support directly and positively influenced the PERMA+4 dimensions of relationships (β = 0.467, *p* = 0.000), meaning (β = 0.355, *p* = 0.000), mindset (β = 0.419, *p* = 0.000), environment (β = 0.230, *p* = 0.008), and economic security (β = 0.155, *p* = 0.036). In addition, positive emotions were influenced by the PERMA+4 dimensions of engagement (β = 0.190, *p* = 0.000), meaning (β = 0.546, *p* = 0.000), accomplishment (β = 0.179, *p* = 0.000), and mindset (β = 0.336, *p* = 0.000). On the other hand, job satisfaction was influenced by positive emotions (β = 0.797, *p* = 0.000) and organizational support (β = 0.293, *p* = 0.000). Finally, organizational commitment was influenced by mindset (β = 0.284, *p* = 0.001), organizational support (β = 0.190, *p* = 0.029), and job satisfaction (β = 0.731, *p* = 0.000).

Regarding the effect size of the relationships found between latent constructs, as can be seen by analyzing the magnitude of the β coefficients, the relationships established between the predictor variables of self-efficacy and organizational support and the criterion dimensions of the PERMA+4 model had a small to moderate effect size, with β coefficients ranging from 0.155 to 0.467. Similarly, the β coefficients obtained for the PERMA+4 dimensions and their relationship with positive emotions determined a small to medium effect size, with values for the β coefficients ranging from 0.179 to 0.336, with the exception of the β coefficient found between the dimension of meaning and positive emotions, which determined a large effect size (β = 0.546). Finally, the relationships found between positive emotions and job satisfaction, and between job satisfaction and organizational commitment indicate a large effect size, with both β coefficients greater than 0.50. The effect size of the relationship between organizational support and job satisfaction was medium, while the effect size corresponding to the relationship between organizational support and organizational commitment was small. In addition, the relationship between mindset and organizational commitment also obtained a medium effect size.

Similarly, the *R*^2^ values obtained for the model’s endogenous variables indicate the existence of small to large effect sizes (Figure 2). For example, self-efficacy alone explained between 10% and 22% of some dimensions of the PERMA+4 model (engagement, accomplishment, and physical health). Organizational support alone explained 31% of the variance of the relationship dimension. Self-efficacy and organizational support jointly explained between 16% and 47% of the variance of some PERMA+4 dimensions (meaning, mindset, environment, and economic security). In turn, the PERMA+4 dimensions of engagement, meaning, accomplishment, and mindset explained 66% of the variance of positive emotions. Finally, the dimensions of positive emotions and organizational support explained almost 90% of the variance of job satisfaction, while 89% of the variance of organizational commitment was explained by the variables of job satisfaction, mindset, and organizational support.

Overall, the results indicate that self-efficacy exerted a positive and significant effect on key dimensions of the PERMA+4 model, such as engagement, meaning, mindset, and accomplishment. This suggests that employees with a strong belief in their own capabilities not only tend to be more engaged in their tasks, but also find greater meaning in their work and develop a greater sense of accomplishment.

On the other hand, perceived organizational support showed a positive association with PERMA+4 dimensions such as relationships at work, meaning, and mindset. In practical terms, this indicates that when employees perceive that the organization values their efforts and cares about their well-being, they are more likely to develop strong work bonds and a growth mindset.

Likewise, the results indicate that the dimensions of engagement, meaning, accomplishment, and mindset had a significant impact on positive emotions. Specifically, meaning and mindset were the variables with the greatest impact on positive emotions, followed by engagement and accomplishment. The combination of these factors explained a considerable proportion of the variability in the positive emotions experienced at work, thus reflecting a strong relationship between job performance and emotional well-being.

In addition, the structural model indicates that positive emotions exerted a significant effect on job satisfaction. This suggests that a substantial part of the variance in job satisfaction is determined by the frequency with which employees experience positive emotions in their work environment. In turn, job satisfaction is the strongest predictor of organizational commitment, indicating that most of the variability in organizational commitment can be attributed to the levels of satisfaction employees reported in their work.

Finally, Table 4 shows the decomposition of the results into direct, indirect, and total effects. The direct effects are the same as those shown in Figure 2 for the relationships between contiguous variables in the model. Indirect effects correspond to all combinations of paths between two latent variables and one or more mediating variables (e.g., the indirect effect of organizational support on organizational commitment through mindset and job satisfaction as mediating variables). Total effects are the sum of the direct and indirect effects that one variable exerts on another variable (R. B. Kline, 2023).

As can be seen in the decomposition of the effects, the results show that self-efficacy and organizational support significantly influenced positive emotions, job satisfaction, and organizational commitment. More specifically, self-efficacy indirectly influenced positive emotions through the mediating variables of engagement, meaning, accomplishment, and mindset (β = 0.427, 95% CI [0.204, 0.656]). Similarly, self-efficacy also indirectly influenced job satisfaction through the mediating variables of engagement, meaning, accomplishment, mindset, and positive emotions (β = 0.340, 95% CI [0.155, 0.552]). In the same way, self-efficacy indirectly influenced organizational commitment through the mediating variables of engagement, meaning, accomplishment, mindset, positive emotions, and job satisfaction (β = 0.249, 95% CI [0.064, 0.523]).

Regarding the relationship found between organizational support, positive emotions, job satisfaction and organizational commitment, the results show in this case that organizational support influenced positive emotions indirectly through the mediating variables of meaning and mindset (β = 0.335, 95% CI [0.188, 0.506]). On the other hand, the results also suggest that, in addition to the direct effects exerted by organizational support, this variable had an indirect influence on job satisfaction and organizational commitment. Thus, organizational support exerted an indirect influence on job satisfaction through the mediating variables of meaning, mindset, and positive emotions (β = 0.267, 95% CI [0.147, 0.418]). Finally, organizational support also indirectly influenced organizational commitment through the mediating variables of meaning, mindset, positive emotions, and job satisfaction (β = 0.528, 95% CI [0.226, 0.816]). As can be seen, organizational support constitutes a relevant variable, as its total effects on job satisfaction (β = 0.559, 95% CI [0.386, 0.754]) and organizational commitment (β = 0.718, 95% CI [0.521, 0.908]) are considerable.

In general, the results of the mediation analyses carried out show that the effects of self-efficacy and organizational support act mainly on the dimensions of the PERMA+4 model, which in turn act on positive emotions and these, ultimately, provide the greatest influence on job satisfaction and organizational commitment. However, the effects that self-efficacy and organizational support generate on some of the PERMA+4 dimensions also extend to positive emotions, job satisfaction, and organizational commitment. Therefore, these findings suggest that self-efficacy and organizational support may lead to an enhancement in the individual’s positive work functioning, which will increase the experience of positive emotions in the work environment, generating a positive influence on job satisfaction and organizational commitment.

## 4. Discussion

The primary aim of this study was to analyze various individual predictors, such as self-efficacy, and organizational factors, such as organizational support, and their influence on workplace well-being, measured through job satisfaction and organizational commitment, within the framework of the PERMA+4 model.

### 4.1. The Direct Impact of Self-Efficacy and Organizational Support

The results support Hypothesis 1, demonstrating that self-efficacy has a significantly positive impact on seven out of the nine dimensions of the PERMA+4 model, confirming its close relationship with workplace well-being. Specifically, self-efficacy is associated with improvements in engagement, meaning, accomplishment, physical health, mindset, environment, and economic security, reinforcing its role as a key psychological resource that promotes not only effective job performance, but also holistic well-being in the workplace. The particular impact of self-efficacy on physical health and meaning underscores the interaction between psychological and biological factors in fostering well-being (Cabrera & Donaldson, 2024; Donaldson et al., 2020; Seligman, 2018). This evidence suggests that employees with high self-efficacy may be better equipped to manage stress, adopt effective self-care strategies, and maintain a healthy balance between personal and professional goals. Furthermore, self-efficacy appears to foster a sense of purpose and meaning at work, strengthening employees’ emotional connection to their tasks and work environment. This aligns with the Psychological Capital model (Luthans et al., 2007), which highlights the importance of psychological resources (self-efficacy, hope, and optimism) in maintaining effective performance and overall well-being.

Regarding organizational support, the findings also confirm that it has a significantly positive effect on five dimensions of the PERMA+4 model (relationships, meaning, mindset, environment, and economic security), validating Hypothesis 2. First, organizational support fosters high-quality workplace relationships based on trust and respect, strengthening emotional bonds among colleagues and purpose in performed tasks. According to Donaldson et al. (2020), this strengthening creates a virtuous cycle that enhances both work engagement and emotional well-being. Additionally, organizational support influences mindset and environment by promoting a growth mindset and an enriching workplace, which, combined with a positive attitude toward learning and self-improvement, stimulate creativity and resilience (Donaldson et al., 2022). Lastly, organizational support directly impacts economic security by providing financial stability, which significantly reduces stress associated with financial concerns and fosters positive emotions such as gratitude and calmness (Donaldson & Donaldson, 2021a).

Moreover, this study confirms organizational support as a direct predictor of job satisfaction (Hypothesis 3) and organizational commitment (Hypothesis 4). Regarding the relationship between organizational support and job satisfaction, the findings suggest that employees who perceive that their contributions are valued and receive emotional and structural backing from the organization exhibit significantly higher levels of job satisfaction. A meta-analysis by Kurtessis et al. (2015) confirms that employees who feel the organization cares about their well-being and recognizes their efforts experience greater satisfaction in their job performance. Similarly, Eisenberger and colleagues (2020) highlight that this perception of organizational support fosters an environment characterized by trust and psychological safety, reducing stress and strengthening intrinsic motivation. Complementarily, Donaldson et al. (2020) emphasize that organizational support also contributes to creating a cohesive and collaborative organizational climate, promoting a more positive and enriching work experience.

Concerning the relationship between organizational support and organizational commitment, other studies have also found that higher organizational support is associated with greater levels of organizational commitment, highlighting that perceived support from the organization directly influences employees’ emotional identification, loyalty to the institution, and their resilience to burnout (Ahmed et al., 2015; Eisenberger et al., 1990; Rhoades et al., 2001). Additionally, this perception of support fosters employees’ retention and compliance with their roles (Eisenberger et al., 2002).

### 4.2. The Influence of the PERMA+4 Model Blocks

Regarding the influence of the PERMA+4 blocks of subjective organizational well-being, several hypotheses were proposed. First, the findings partially confirm Hypothesis 5, which states that the PERMA+4 blocks of Engagement, Relationships, Meaning, Accomplishment, Physical Health, Mindset, Environment, and Economic Security will positively affect the PERMA+4 block of Positive Emotions. The results of this study confirm that only the blocks of engagement, meaning, accomplishment, and mindset significantly impact positive emotions, a central component of workplace well-being.

However, other dimensions, such as relationships, physical health, and environment, did not show a significant effect on positive emotions. This could be influenced by contextual factors, such as the specific characteristics of the sample or the organizational setting. In workplaces where employees already perceive stable relationships or a satisfactory physical environment, these dimensions may not generate noticeable variations in emotional well-being (Fernández-López et al., 2010; Gu et al., 2022). Additionally, individual differences, such as employees’ baseline health status or their working conditions, could moderate these effects, limiting their impact on positive emotions (Kurtessis et al., 2015). Methodologically, the cross-sectional nature of the study may also have played a role, as some relationships might require a longer time frame to manifest. Future research using longitudinal designs could provide further insights into the long-term effects of these dimensions on workplace well-being.

Engagement facilitates flow states, where balance is achieved between skills and job challenges, generating immediate positive emotions such as satisfaction and enthusiasm (Csikszentmihalyi, 2000; Donaldson et al., 2022). Meaning contributes to emotional well-being by generating pride and satisfaction among employees who perceive their efforts as having transcendent value (Seligman, 2018; Donaldson et al., 2020). Similarly, accomplishment enhances emotions such as gratitude, optimism, and satisfaction while increasing employees’ confidence in their abilities (Donaldson et al., 2022; Fredrickson, 2001). Finally, mindset fosters resilience and optimism, helping employees view challenges as opportunities for learning rather than obstacles, thereby strengthening constructive emotions and overall well-being (Dweck, 2008; Donaldson et al., 2022).

Continuing with the influence of the PERMA+4 model, the results confirm an integrated system in which each of its blocks contributes specifically to job satisfaction and organizational commitment. Regarding Hypothesis 6, which posits that the PERMA+4 blocks will have positive effects on Job Satisfaction and Organizational Commitment, the findings partially confirm this. Only mindset has a direct effect on organizational commitment. This result, consistent with Dweck’s (2008) studies, indicates that a positive mindset enables employees to face challenges and align with organizational values, reinforcing their sense of belonging and loyalty to the organization.

This result suggests that organizational commitment may be less influenced by immediate emotional states or environmental factors and more dependent on cognitive and attitudinal variables, such as an employee’s mindset (Rego et al., 2012). This aligns with prior research indicating that resilience, optimism, and a proactive approach to challenges contribute more significantly to long-term commitment than transient emotional experiences (Dweck, 2008). The absence of significant effects for other PERMA+4 dimensions could also be due to methodological factors. Since commitment is a construct that tends to develop over time, a cross-sectional study may not fully capture its evolution.

### 4.3. Positive Emotions as a Pathway to Job Satisfaction

The impact of PERMA+4 dimensions (engagement, meaning, accomplishment, and mindset) on job satisfaction is indirect, mediated through positive emotions (Hypothesis 7). However, in this study, these positive emotions do not directly influence organizational commitment, suggesting their effects are limited to job satisfaction.

This mechanism confirms the fundamental role of positive emotions as a crucial process for transforming positive workplace experiences into job satisfaction (Fredrickson, 2001; Seligman, 2018). Engagement facilitates the experience of flow, a state where job challenges and skills are balanced, generating immediate positive emotions that contribute to increased job satisfaction (Csikszentmihalyi, 2000; Schaufeli et al., 2002). Additionally, when employees perceive that their work has meaning or achieve accomplishments, they experience positive emotions such as pride, gratitude, and satisfaction, which enhance both workplace well-being and job satisfaction (Pratt & Ashforth, 2003; Steger et al., 2006). These experiences also strengthen employees’ confidence in their abilities, promoting self-efficacy (Bandura, 1997). According to Dweck (2008), a growth mindset fosters resilience and a readiness to face challenges, generating positive emotions as employees perceive work as an opportunity for continuous development (Luthans et al., 2007; Donaldson et al., 2020). Finally, based on Fredrickson’s Broaden and Build theory (2001), positive emotions broaden employees’ cognitive and emotional perspectives, allowing them to build personal resources that help them better navigate workplace challenges. These emotions create a favorable emotional environment that increases job satisfaction (Connolly & Viswesvaran, 2000).

### 4.4. The Mediating Role of Positive Emotions in the Relationship Between Model Blocks and Job Satisfaction

In this way, Hypothesis 8, which proposed that positive emotions mediate the relationship between PERMA+4 dimensions and Job Satisfaction and Organizational Commitment, is partially confirmed. Positive emotions mediate only the relationship between PERMA+4 dimensions and job satisfaction, acting as a mediating mechanism that translates positive experiences into higher job satisfaction. However, organizational commitment exhibits a different relationship, as it depends exclusively on the direct effects of mindset. This concept, which encompasses resilience, optimism, and a readiness to learn, fosters a deep emotional connection with the organization (Dweck, 2008).

The findings of this study highlight that job satisfaction and organizational commitment are influenced by distinct mechanisms, requiring specific interventions to address and optimize both constructs. While strategies aimed at fostering self-efficacy, organizational support, and positive emotions may be highly effective for increasing short-term job satisfaction, organizational commitment demands a more sustained approach over time. This approach should focus on developing mindset, strengthening organizational support, and enhancing job satisfaction, which emerges as its primary predictor. Job satisfaction increases employees’ emotional identification and loyalty toward the organization, promoting a sense of belonging and a greater willingness to actively contribute to organizational success (Brooke et al., 1988; Eisenberger et al., 2020; Locke, 1969). This combined approach not only enhances employee satisfaction and commitment, but also contributes to the organization’s sustainable success by aligning individual experiences with collective goals.

This study makes it clear that satisfaction and commitment are distinct processes, although they are closely related. Companies that successfully balance both aspects will not only have happier employees, but also stronger, more resilient teams that are aligned with organizational goals. One of the first actions that can make a significant difference is strengthening employees’ confidence in their own abilities. Self-efficacy has been shown to be a key determinant, and fostering it does not require large investments. It is enough to create spaces where employees receive constructive feedback, have opportunities to develop new skills, and can take on responsibilities that challenge and motivate them.

However, individual confidence is not built in isolation. Organizational support plays a crucial role in shaping how employees perceive their place within the company. Feeling supported by the organization not only impacts job satisfaction, but also influences motivation and the willingness to commit to company objectives. Here, leaders play a fundamental role. Their responsibility is not just to manage teams but to be present, listen, and recognize each person’s efforts. A leadership style based on closeness and genuine support can completely transform the work experience. Additionally, offering flexibility, economic stability, and benefits that facilitate work-life balance strengthens this perception of support and reduces work-related stress.

For these initiatives to have a lasting impact, well-being must be approached holistically. The PERMA+4 model provides a useful framework for understanding how different dimensions contribute to a more fulfilling work experience. Small actions can drive big changes: creating spaces for collaboration and teamwork to strengthen interpersonal relationships, ensuring a comfortable physical work environment with natural light and basic amenities, fostering a corporate culture that values personal and professional growth, and ensuring that each employee finds a sense of purpose in their work.

That said, improving job satisfaction is not enough if the goal is to build genuine, long-term organizational commitment. While aspects such as self-efficacy, organizational support, and positive emotions can boost short-term satisfaction, true commitment requires a deeper vision. This is where the development of a growth mindset becomes essential, enabling employees to not only feel good in their current roles, but also envision a future within the organization. Providing continuous learning opportunities, designing clear career paths, and fostering a culture where challenges are seen as opportunities rather than obstacles can make the difference between an employee who is merely satisfied with their job and one who truly feels part of the company in the long run.

### 4.5. Limitations and Future Research Directions

The cross-sectional nature of the data does not allow for definitive causal relationships to be established, making it impossible to confirm the direction of these relationships. This limitation affects the understanding of how interventions based on the PERMA+4 model might influence workplace well-being over time. Future research should adopt longitudinal designs to examine the long-term effects of such strategies, providing a more comprehensive view of their impact on employee well-being and organizational success.

Another factor to consider is individual differences, such as gender, age, or educational level, which might act as moderators of the observed relationships. For instance, previous research has found that there is a positive relationship between worker age and job satisfaction (Cavanagh et al., 2020). This association can be explained by the fact that, as the person ages, the characteristics of the job that are attractive and motivating change and, therefore, the determinants of job satisfaction are modified. These differences could influence the effectiveness of proposed interventions, making it essential to understand them in order to design programs tailored to the specific needs of different groups.

Finally, it is necessary to account for various economic, organizational, and cultural contexts. These contexts may shape how employees experience workplace well-being, making it crucial to expand research to diverse settings to determine whether the observed relationships are generalizable or if the model needs adaptation to different contexts. Additionally, the use of snowball sampling may have introduced biases in participant selection, potentially limiting the diversity of the sample and affecting the generalizability of the findings. Future research should consider more representative sampling techniques to enhance the external validity of the results.

## 5. Conclusions

This study examined the role of self-efficacy and organizational support in workplace well-being, using the PERMA+4 framework as a reference model. Findings indicate that self-efficacy is a key predictor of multiple dimensions of well-being, particularly engagement, meaning, and accomplishment, reinforcing its relevance as a psychological resource that enhances job satisfaction and organizational commitment.

Similarly, organizational support emerged as a significant factor, fostering high-quality work relationships, a growth-oriented mindset, and economic security. These elements contribute to a more stable and fulfilling work environment, underscoring the importance of organizational efforts to enhance perceived support and promote a positive workplace culture.

The results confirm that positive emotions mediate the relationship between workplace experiences and job satisfaction, emphasizing the role of engagement, meaning, and accomplishment in fostering emotional well-being. Given the distinct pathways influencing job satisfaction and commitment, tailored interventions that strengthen self-efficacy, mindset, and organizational support may be particularly effective in improving employee well-being and retention.

Moreover, the findings of this study demonstrate that job satisfaction and organizational commitment require complementary approaches: while short-term well-being is strengthened through the development of self-efficacy and organizational support, employee retention and loyalty depend on long-term strategies that reinforce their sense of purpose and growth. Companies that invest in employee development, foster close and supportive leadership, and create an environment based on the principles of the PERMA+4 model not only enhance the daily work experience, but also build an organizational culture where people feel valued and motivated.

## Figures and Tables

**Table 1:** Summary of the results of the confirmatory factor analysis (CFA).

Factors and Items	λ	α
1. Self-efficacy		0.797
Confío en mis propias capacidades en situaciones difíciles en el trabajo.	0.749	
Soy capaz de resolver la mayoría de los problemas en mi trabajo por mí mismo.	0.867	
Normalmente puedo resolver bien incluso tareas difíciles y complejas en mi trabajo.	0.629	
2. Organizational support		0.880
Mi supervisor/a me ayuda en la realización de mi trabajo.	0.724	
Mi supervisor/a presta atención a lo que le digo.	0.782	
Mi supervisor/a se preocupa por el bienestar de sus trabajadores.	0.847	
Me siento apreciado por mi supervisor/a.	0.867	
3. Positive emotions		0.897
Me siento contento/a en un día típico de trabajo.	0.796	
En general, me siento entusiasmado/a con mi profesión.	0.886	
Me gusta mi trabajo.	0.922	
4. Engagement		0.839
Me suelo quedar absorto/a mientras trabajo en aquello que desafía mis habilidades.	0.747	
Mientras hago algo que me gusta en el trabajo, pierdo la noción del tiempo.	0.894	
Cuando estoy trabajando en algo que me gusta, me olvido de todo lo que me rodea.	0.755	
5. Relationships		0.904
Puedo recibir apoyo de mis compañeros/as de trabajo si lo necesito	0.780	
Me siento valorado/a por mis compañeros/as de trabajo.	0.882	
Confío en mis colegas de trabajo.	0.883	
Mis colegas de trabajo sacan lo mejor de mí.	0.822	
6. Meaning		0.877
Mi trabajo tiene sentido.	0.879	
Entiendo aquello que hace que mi trabajo tenga sentido.	0.917	
El trabajo que hago sirve a un propósito mayor.	0.746	
7. Accomplishment		0.843
Me fijo metas que me ayudan a lograr mis aspiraciones profesionales.	0.699	
Por lo general cumplo lo que me propongo en mi trabajo.	0.881	
En general, estoy satisfecho/a con mi rendimiento en el trabajo.	0.855	
8. Physical health		0.814
En general, me siento físicamente saludable.	0.737	
Rara vez estoy enfermo/a.	0.655	
Normalmente puedo superar las situaciones de malestar físico (insomnio, lesiones y problemas de visión).	0.642	
Siento que controlo mi salud física.	0.860	
9. Mindset		0.725
Creo que puedo mejorar mis habilidades laborales trabajando duro.	0.524	
Creo que mi trabajo me permitirá desarrollarme en el futuro.	0.727	
Tengo un futuro brillante en la organización en la que trabajo actualmente.	0.816	
10. Environment		0.875
El entorno físico de trabajo (por ejemplo, espacio en la oficina) me permite concentrarme en mi trabajo.	0.926	
Hay mucha luz natural en mi lugar de trabajo.	0.913	
Puedo acceder con facilidad a la naturaleza en mi entorno de trabajo (por ejemplo, parques, playas y montañas).	0.702	
11. Economic security		0.761
Me siento cómodo/a con mis ingresos actuales.	0.555	
Podría perder varios meses de sueldo por una enfermedad grave y seguir teniendo mi seguridad económica.	0.829	
En caso de una emergencia financiera, tengo suficientes ahorros.	0.804	
12. Job satisfaction		0.822
No estoy contento/a con mi trabajo.	0.711	
Con frecuencia me aburro en mi trabajo.	0.566	
Disfruto con mi trabajo.	0.830	
La mayoría de los días estoy entusiasmado/a con mi trabajo.	0.843	
13. Organizational commitment		0.826
Me siento parte de esta empresa.	0.753	
Incluso si esta organización no marchara bien, sería reacio/a a cambiar de organización.	0.571	
En mi trabajo, me gusta sentir que estoy esforzándome no sólo por mí, sino también por mi organización.	0.615	
Estoy muy orgulloso/a de decirle a la gente la empresa en la que trabajo.	0.749	
Me complace saber que mi trabajo ha contribuido al bien de la empresa.	0.800	

Note. λ = factor loadings of each item; α = Cronbach’s α.

**Table 2:** Correlations between latent variables and construct validity and reliability indices.

	CR	AVE	MSV	1	2	3	4	5	6	7	8	9	10	11	12	13
1. Self-efficacy	0.785	0.566	0.297	**0.752**												
2. Organizational support	0.875	0.647	0.566	0.510	**0.804**											
3. Positive emotions	0.909	0.760	0.584	0.428	0.434	**0.872**										
4. Engagement	0.844	0.644	0.213	0.239	0.226	0.461	**0.802**									
5. Relationships	0.909	0.710	0.281	0.342	0.530	0.390	0.218	**0.843**								
6. Meaning	0.883	0.710	0.584	0.443	0.499	0.764	0.355	0.442	**0.843**							
7. Accomplishment	0.850	0.648	0.259	0.348	0.253	0.509	0.249	0.282	0.467	**0.805**						
8. Physical health	0.822	0.546	0.181	0.406	0.268	0.315	0.158	0.257	0.323	0.426	**0.739**					
9. Mindset	0.732	0.509	0.480	0.498	0.575	0.682	0.380	0.381	0.619	0.386	0.332	**0.713**				
10. Environment	0.874	0.702	0.255	0.267	0.323	0.369	0.209	0.248	0.401	0.298	0.198	0.505	**0.838**			
11. Economic security	0.797	0.564	0.188	0.364	0.304	0.275	0.050	0.233	0.255	0.184	0.248	0.434	0.318	**0.751**		
12. Job satisfaction	0.819	0.542	0.460	0.490	0.628	0.711	0.421	0.490	0.640	0.469	0.303	0.693	0.442	0.300	**0.736**	
13. Organizational commitment	0.826	0.503	0.460	0.545	0.752	0.651	0.374	0.481	0.602	0.373	0.325	0.600	0.467	0.397	0.678	**0.709**

Note. AVE = average variance extracted; MSV = maximum shared variance. Bold values on the diagonal correspond to the square root of the AVE.

**Table 3:** Discriminant validity through the HTMT2 ratio (heterotrait–monotrait).

	1	2	3	4	5	6	7	8	9	10	11	12
1. Self-efficacy												
2. Organizational support	0.472											
3. Positive emotions	0.407	0.442										
4. Engagement	0.201	0.218	0.455									
5. Relationships	0.351	0.512	0.420	0.233								
6. Meaning	0.421	0.484	0.783	0.362	0.434							
7. Accomplishment	0.360	0.245	0.554	0.283	0.304	0.513						
8. Physical health	0.424	0.266	0.333	0.170	0.256	0.332	0.419					
9. Mindset	0.395	0.586	0.708	0.419	0.400	0.658	0.446	0.360				
10. Environment	0.208	0.290	0.377	0.237	0.247	0.374	0.309	0.168	0.465			
11. Economic security	0.347	0.363	0.346	0.068	0.277	0.297	0.202	0.254	0.392	0.367		
12. Job satisfaction	0.413	0.588	0.722	0.382	0.501	0.747	0.480	0.303	0.553	0.401	0.346	
13. Organizational commitment	0.520	0.730	0.770	0.371	0.470	0.724	0.404	0.329	0.649	0.448	0.472	0.760

**Table 4:** Direct, indirect, and total effects for the relationships among the study variables.

Outcome Variable	Predictor	Effects Decomposition		
		Direct	Indirect (95% LLCI, ULCI)	Total (95% LLCI, ULCI)
Positive Emotions	Engagement	0.190 ***	-	-
	Meaning	0.546 ***	-	-
	Accomplishment	0.179 ***	-	-
	Mindset	0.336 ***	-	-
	Self-efficacy	-	0.427 (0.204, 0.656)	-
	Organizational Support	-	0.335 (0.188, 0.506)	-
Job Satisfaction	Engagement	-	0.152 (0.078, 0.231)	-
	Meaning	-	0.435 (0.310, 0.573)	-
	Accomplishment	-	0.143 (0.074, 0.220)	-
	Mindset	-	0.268 (0.139, 0.415)	-
	Self-efficacy	-	0.340 (0.155, 0.552)	-
	Organizational Support	0.293 ***	0.267 (0.147, 0.418)	0.559 (0.386, 0.754)
	Positive Emotions	0.797 ***	-	-
Organizational Commitment	Engagement	-	0.111 (0.034, 0.220)	-
	Meaning	-	0.318 (0.111, 0.572)	-
	Accomplishment	-	0.104 (0.030, 0.217)	-
	Mindset	0.284 **	0.196 (0.060, 0.392)	0.480 (0.259, 0.738)
	Self-efficacy	-	0.249 (0.064, 0.523)	-
	Organizational Support	0.190 *	0.528 (0.226, 0.816)	0.718 (0.521, 0.908)
	Positive Emotions	-	0.583 (0.212, 0.801)	-
	Job Satisfaction	0.731 ***	-	-

Note. * *p* < 0.05; ** *p* < 0.01; *** *p* < 0.001; LLCI = lower-level confidence interval; ULCI = upper-level confidence interval.

## Data Availability

The data presented in this study are available on request from the corresponding author. The data are not publicly available due to standard privacy and confidentiality considerations.
